# Ejaculate Ejection by Female Fruit Flies Does Not Correlate With Mating Latency or Male Presence, Irrespective of How Ejection Is Measured

**DOI:** 10.1002/ece3.72281

**Published:** 2025-10-07

**Authors:** Krish Sanghvi, Biliana Todorova, Irem Sepil

**Affiliations:** ^1^ Department of Biology University of Oxford Oxford UK

**Keywords:** cryptic female choice, mate choice, mating plug, sexual conflict, sexual selection, sperm ejection

## Abstract

Sexual selection operates across pre‐ and post‐mating episodes, driven by intra‐sexual competition and inter‐sexual choice for mates or gametes. While these episodes can act in the same direction, this is not always the case; for example, males who are preferred as mates by females may produce sperm not preferred for fertilisation. In many species, post‐mating sexual selection is measured using ejaculate ejection behaviour. However, whether different proxies for this behaviour correlate with each other, and whether the environment experienced by females post‐mating influences ejaculate ejection, has received little attention until recently. Using *Drosophila melanogaster* , we first explore whether mating latency, a proxy for inter‐sexual pre‐mating preference, correlates with different proxies for female post‐mating preference (ejection latency, ejection likelihood, numbers of sperm ejected, numbers of sperm retained and proportion of sperm ejected). We further test whether the presence of a male in the female's post‐mating environment influences such ejaculate ejection behaviour. We find no effect of male presence. Additionally, mating latency does not correlate with any measure of ejaculate ejection behaviour, possibly indicating the decoupling of sexual selection at pre‐ and post‐mating stages. Importantly, we find that ejection latency, commonly used to quantify female post‐mating preference, does not correlate with the numbers of sperm ejected or retained, or the proportion of sperm ejected. Instead, females that eject more sperm also retain more sperm due to variation in sperm numbers inseminated by males. This highlights the importance of using different proxies for ejaculate ejection behaviour in tandem and carefully interpreting their biological meaning.

## Graphical abstract

How to measure female ejaculate ejection, is it related to pre‐mating sexual selection, or influenced by post‐mating sexual environment? We explore these questions in fruit flies.

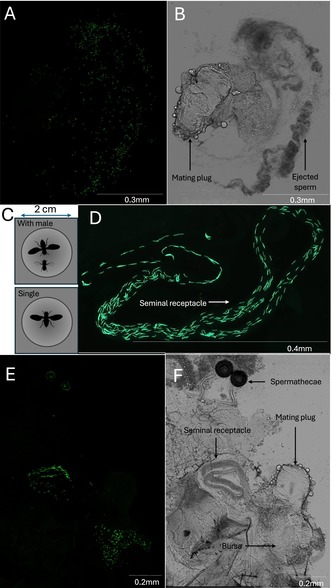

## 1 Introduction

Sexual selection occurs at both pre‐ and post‐mating stages, each partitioned into intra‐sexual competition between mates and inter‐sexual preference for gametes (Kuijper et al. 2012; G. A. Parker 2014; Pizzari et al. 2002; Shuker and Kvarnemo 2021). Females commonly mediate sexual selection before and after mating via preference for specific mates or their sperm, respectively. In females, pre‐mating preference is typically expressed as choosing to mate with males that have more elaborate phenotypes (Dougherty 2020; Edward 2015; Edward and Chapman 2011) and accepting matings from attractive males more quickly (Davies et al. 2020; Reinhold and Schielzeth 2015). On the other hand, female post‐mating preference, which allows females to bias fertilisation towards certain males (Eberhard 2015; Firman et al. 2017), is in many species measured as ejaculate ejection behaviour (e.g., Pizzari and Birkhead 2000). The combined effects of both pre‐ and post‐mating sexual selection determine male and female reproductive success (Hunt et al. 2009; Lüpold et al. 2020).

Episodes of pre‐ and post‐mating sexual selection can be female‐ and/or male‐driven and can act synergistically (Devigili et al. 2015; Marie‐Orleach et al. 2024; McDonald et al. 2017; Sanghvi, Henshaw, et al. 2025). For example, a male preferred as a mate due to having ‘sexier’ ornamentation might also be preferred post‐mating, whereby females retain more of his sperm for fertilisation when the preference is female‐driven (e.g., D. Parker 2009; Sbilordo and Martin 2014). Alternatively, males in higher condition that are better at acquiring mates due to having larger weaponry might also produce larger or more competitive ejaculates if the synergy is male‐driven (e.g., Durrant et al. 2016; Locatello et al. 2006; Puniamoorthy et al. 2012). On the other hand, pre‐ and post‐mating episodes of sexual selection can act disruptively. Here again, females can influence outcomes, for example, by ejecting a greater proportion of sperm from males that are more successful at coercing them into mating (Dean et al. 2011). Alternatively, males can drive the outcome if they face energetic trade‐offs toward allocation in traits that facilitate mate acquisition as opposed to ejaculate traits; or conversely, if less ‘sexy’ males produce larger or more competitive ejaculates (Danielsson 2001; Engqvist 2011; Evans and Garcia‐Gonzalez 2016; Ferrandiz‐Rovira et al. 2014; Kim and Velando 2020; Sanghvi, Henshaw, et al. 2025). Therefore, male and female‐driven processes can act in concert in both pre‐ and post‐mating contexts, shaping the outcome of sexual selection. Correlations between proxies for pre‐ and post‐mating sexual selection can be used to assess whether they operate in the same or opposite directions (Kvarnemo and Simmons 2013; Pilastro et al. 2004). However, the biological relevance of such correlations remains uncertain, as studies often rely on a single proxy for post‐mating sexual selection, such as ejaculate ejection latency (Lüpold et al. 2013; Manier et al. 2010; Yun et al. 2024), the number of sperm ejected or retained (Córdoba‐Aguilar 2006; Doubovetzky et al. 2024), or the likelihood of ejaculate ejection (Pizzari and Birkhead 2000). However, different proxies (listed in Eberhard 2015; Firman et al. 2017) might have different biological mechanisms influencing them and thus might not be interchangeable. Furthermore, in specific socio‐sexual environments, some proxies might be better indicators of post‐mating sexual selection than others. It is therefore crucial to test how both the choice of proxy and the socio‐sexual environment influence the correlation between pre‐ and post‐mating sexual selection; however, this has received little attention.

The fruit fly, *Drosophila melanogaster* , provides an excellent model to address these gaps. Male fruit flies perform elaborate courtship displays that females assess before mating (Pan et al. 2011; Pavlou and Goodwin 2013). Mating latency is a reliable and commonly used indicator for female pre‐mating preference, with males in higher condition or better at courtship typically having shorter latencies (e.g., Dewan et al. 2025; Dukas 2005; Hosken et al. 2008; Poissonnier et al. 2024; Savic Veselinovic et al. 2017; Sharma et al. 2010). Post‐mating, females store sperm in the seminal receptacle and spermathecae, and eject any sperm not stored in these storage organs, along with the male's ‘mating plug’ within a few hours (Manier et al. 2010; Schnakenberg et al. 2012). This ‘mating plug’ is made of male‐ and female‐derived proteins, which temporarily prevents female re‐mating, thereby reducing sperm competition (Avila et al. 2015; Bretman et al. 2010; McDonough‐Goldstein et al. 2022). Females can control the timing of ejaculate ejection (Laturney and Billeter 2016; Lee et al. 2015; Lüpold et al. 2013; Mahdjoub et al. 2023; Schnakenberg et al. 2012) based on the mated male's relative quality to other males by removing the mating plug. For instance, females eject ejaculates sooner when they can smell the cuticular pheromones of more attractive males in their post‐mating environment, compared to pheromones of less attractive males or these pheromones being absent (Doubovetzky et al. 2024; Yun et al. 2024). Males too can influence female ejaculate ejection behaviour via seminal fluid proteins (Avila et al. 2015). While female ejection latency, commonly used as a metric of her post‐mating preference, is assumed to be correlated, thus interchangeable with the numbers of sperm stored (Doubovetzky et al. 2024; Manier et al. 2010), evidence for this association is lacking (however, see Lüpold et al. 2020). Additionally, whether the environment experienced by females post‐mating influences female ejaculate ejection behaviour, and the dependence of this relationship on the choice of proxy used, remains unclear.

Using *D. melanogaster*, we address three aims. In Aim 1, we test whether pre‐ and post‐mating sexual selection act in the same direction by investigating how mating latency correlates with several measures of ejaculate ejection behaviour. This correlation can either be positive, zero or negative and be male‐ and/or female‐driven. Pre‐ and post‐mating sexual selection can act complementarily, such that males preferred as mates (female‐driven) or males better at courting (male‐driven) also have their sperm preferred for fertilisation (female‐driven) or produce ejaculates that are more difficult to eject or sperm that are better able to enter female sperm‐storage organs (male‐driven). If so, we predict that males with shorter mating latencies will have more of their sperm retained and their ejaculates ejected later by females. On the other hand, if there are trade‐offs between male investment in courtship versus ejaculates (Simmons et al. 2017) (male‐driven) or females eject sperm of more coercive males via cryptic female choice (female‐driven), then we predict that mating latency would correlate negatively with the numbers of sperm ejected and positively with ejection latency. In Aim 2, we investigate how the presence of another male in a female's post‐mating environment influences different proxies of her ejaculate ejection behaviour. Polyandry improves the reproductive success of female *D. melanogaster* (Yan et al. 2025) and provides benefits to females by increasing the genetic diversity of her offspring (Jennions and Petrie 2000). We thus predict that females might eject the first male's ejaculate sooner and in greater amounts in the presence of another male to facilitate mating with this novel male. In fruit flies, earlier ejection could also occur due to pheromones in the mating plug masking female attractiveness to other males (Laturney and Billeter 2016). In Aim 3, we quantify whether different proxies for female ejaculate ejection behaviours correlate with each other to explore whether these are interchangeable and comparable when interpreting post‐mating sexual selection. Here, we predict that if sperm have a constant rate of entering female sperm‐storage organs, then earlier ejection of ejaculates would lead to fewer sperm being retained, therefore more sperm being ejected by females. However, if males vary substantially in the total quantities of ejaculate transferred to females, then those who transfer more sperm may not only have more sperm stored but also more sperm ejected by females, making these proxies un‐interchangeable (Box 1).

BOX 1 **Sperm ejection through a resource partitioning lens.**

A common assumption in the literature is that if females eject more sperm, they should retain fewer, leading to a negative correlation between the number of sperm ejected and retained. Therefore, studies often infer female post‐copulatory preference by measuring a single proxy to quantify ejaculate ejection.

This view is misleading, and the mathematical framework of life‐history theory that considers resource allocation and acquisition provides a means to demonstrate why (Roff and Fairbairn 2007; Van Noordwijk and De Jong 1986). Among‐individual correlations between traits are shaped by variation in resource allocation versus acquisition. If individuals differ more in how they are partitioning resources to each trait, then the traits themselves correlate negatively. However, if individuals differ more in how much resource they acquire, the traits correlate positively. Akin to this life‐history model, sperm ejection behaviour can be represented as:

$$T_{\left[i\right]}=\left(P_{E\left[i\right]}+P_{R\left[i\right]}\right)\cdot T_{\left[i\right]}$$

where $T_{\left[i\right]}$ is the total number of sperm transferred to the *i*th female, $P_{E\left[i\right]}$ is the *proportion* of inseminated sperm that is ejected, and $P_{R\left[i\right]}$ is the *proportion* that is retained. Thus $P_{E\left[i\right]}\cdot T_{\left[i\right]}$ is the *number* of sperm ejected, $P_{R\left[i\right]}\cdot T_{\left[i\right]}$ is the *number* of sperm retained.

Here, greater among‐female variation in the proportion of sperm ejected, relative to variation in the number of sperm inseminated, would result in a negative correlation between the number of sperm retained and the number ejected (Sanghvi, Gascoigne, and Sepil 2025). However, if ‘resource acquisition’, ‘*T*’ (i.e., the total number of sperm inseminated by a male into a female) varies more among females than the proportion of sperm that is ejected (i.e., $P_{E}$ or 1−$P_{R}$), then a positive correlation between these measures would be observed (see Figure S1).

This scenario is problematic, because if a study only assays the numbers of sperm ejected and finds that females eject fewer sperm of males of type ‘A’ than type ‘B’, it does not mean that type ‘A’ males are preferred. Instead, this result is a consequence of type ‘A’ males inseminating fewer sperm overall, which would also lead to type ‘A’ males having fewer sperm retained. Now, if another study only assays the numbers of sperm retained, the opposite conclusion about preference would be reached. Therefore, only measuring either absolute sperm numbers ejected or retained is not informative about post‐mating sexual selection, due to their correlations and biological interpretation being vulnerable to the amount of variation in total sperm inseminated. We therefore suggest that the *proportion* of total sperm ejected or retained ($P_{E}$ or 1−$P_{E}$) should be used concurrently with these other proxies.

## 2 Methods

### 2.1 Stock Maintenance

We used *D. melanogaster* populations from three different lines for our experiment. Focal females in our experiment came from the Dahomey (*dah*) line, focal males came from the (*gfp*) line and ‘competitor’ males (i.e., the male in the female's post‐mating environment) came from the (*sot*) line. Males in the *gfp* line have sperm heads tagged with green fluorescent protein expressed at the *Mst35Ba* and *Mst35Bb* loci, enabling visualisation of these (Manier et al. 2010). *sot* line males produce only seminal fluid, ensuring no sperm transfer in the treatment where females were kept with a competitor male (Sanghvi, Shandilya, et al. 2025). To generate experimental flies, we used a standard developmental density method (following Clancy and Kennington 2001). Experimental flies were collected within 6 h of eclosion to ensure virginity using ice anaesthesia. All flies used in our experiments were between 7 and 11 days old and kept on a 12D:12L cycle at 25°C with ~45% r.h. and fed on Lewis medium supplemented with molasses and *ad libitum* yeast. More details on animal husbandry are presented in Appendix 1.

### 2.2 Experimental Design

#### 2.2.1 Mating Assay

We first mated *dah* females to *gfp* males. For this, virgin *gfp* males were introduced individually into vials, each containing a single virgin *dah* female, and their mating behaviours (mating latency and copulation duration) were recorded via continuous scanning of vials. Once copulation ceased, the mated *dah* female was immediately transferred to a 3D printed black plastic chamber (‘ejection chamber’), of 2 cm in diameter, for ejaculate ejection assays, which was also kept at 25°C and 45% r.h.

#### 2.2.2 Ejection Assay

**FIGURE 1:**
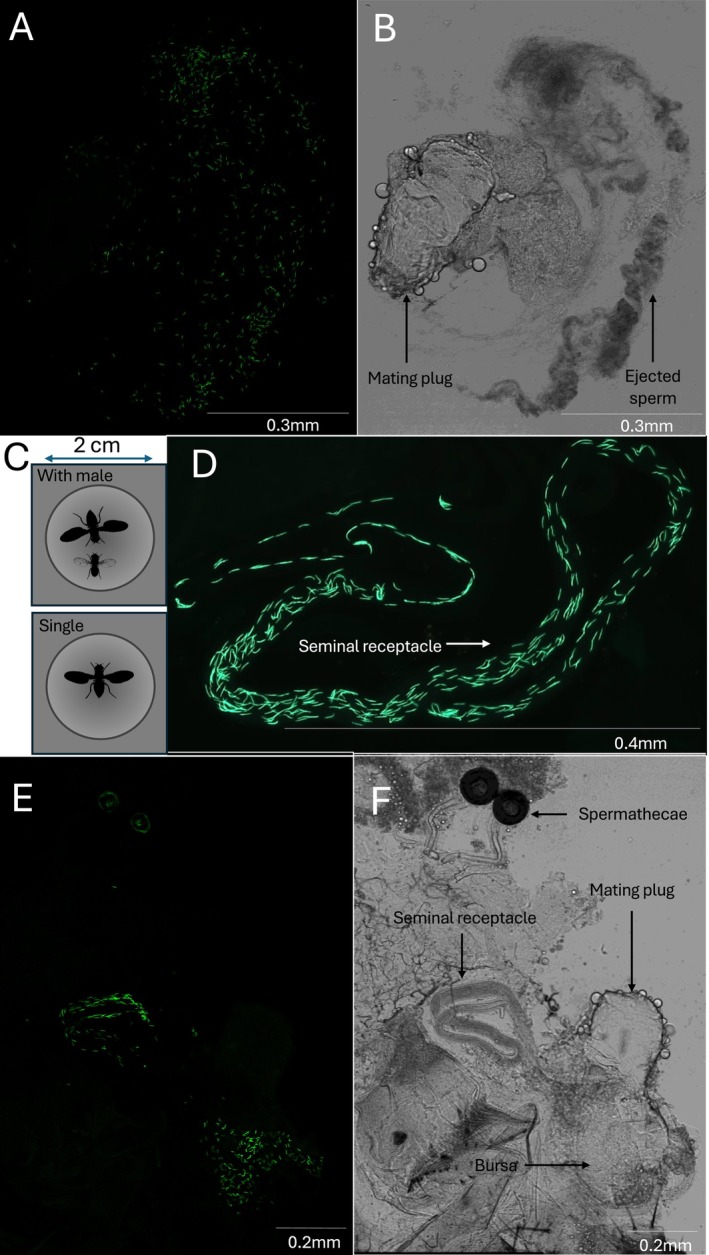
Images of ejected ejaculates and stored sperm, and diagram of ejection chambers. GFP channel (A) and T‐PMT (white light) channel (B) images of the ejaculate ejected by a female. (C) Our two experimental treatments showing females either kept singly or with another male inside the ejection chamber (2 cm inner diameter). (D) Sperm inside the seminal receptacle of a female post ejaculate ejection. GFP (E) and T‐PMT (F) channel images of the reproductive tract of a female who did not eject the ejaculate, thus had mating plug intact and sperm present in the bursa. In A, C, E, green dots/lines show fluorescent sperm heads of individual sperm. Silhouettes taken from PhyloPics are under the CC0 1.0 and Public Domain Mark 1.0 licences.

Mated females in the ejection chambers were assigned to one of two treatments, either being kept singly in the chamber or paired with a virgin *sot* male. Female flies use multiple modalities to detect the presence of males and assess quality (Ishimoto and Kamikouchi 2021), and we did not want to rely on a single modality in our assay. Therefore, unlike Yun et al. (2024) who only ‘simulated’ male presence in the female's post‐mating environment by using male pheromones, we instead used a live male. Once in the ejection chamber, the females were closely and continuously inspected for up to 12 h using a dissecting microscope (20× magnification) for the presence of an ejected mating plug (Figure 1A,B) until ejection occurred. Once an ejected mating plug was observed, it was immediately removed from the chamber (Figure 1C) and mounted for imaging the following day (Appendix 2). Additionally, the female was immediately frozen at −20°C for future dissection and imaging for sperm counts (following Sanghvi, Shandilya, et al. 2025; Appendix 3). For all instances of ejaculate ejections, we recorded the ejection latency. When ejection did not occur, the female was right‐censored. The mating and ejection assays took place over 2 days, with a different set of flies used on each day (Table 1). Only one female was observed re‐mating with the *sot* male, and she was excluded from all analyses.

**TABLE 1:** Sample sizes for different assays in our experiment across the two experimental days and treatments. ‘Mated’—the number of male–female pairs that mated, thus for whom mating latency data was recorded. ‘Ejected’—number of females that ejected ejaculates, thus for whom ejection latency data was recorded. Females who did not eject were right‐censored. ‘Ejected sperm counts’—number of females for whom the ejected sperm numbers were counted. ‘SR sperm counts’—number of females for whom sperm numbers in the seminal receptacle were counted. Due to some females or ejected ejaculate samples being lost, sample sizes are lower in later assays. Note that on Day 1, experimental flies were between 7 and 9 days old and females were transferred into and out of the ejection chambers using an aspirator. On Day 2, experimental flies were between 9 and 11 days old and females were transferred into and out of the ejection chamber using CO_2_ anaesthesia lasting between 5 and 10 s. CO_2_ was used to reduce the number of females that escaped when using an aspirator (which was the case on Day 1).

	Mated	Ejected	Ejected sperm counts	SR sperm counts
Day 1				
Single	17	16	16	15
With male	14	9	9	8
Day 2				
Single	16	14	14	13
With male	19	15	14	13
Total				
Single	33	30	30	28
With male	33	24	23	21

#### 2.2.3 Sperm Imaging and Counts

We dissected the frozen females who ejected part of their ejaculate and counted the number of sperm stored in the seminal receptacle post‐ejection. We also counted the number of sperm in the ejected ejaculate (Appendices 2 and 3 Figure 1D–F). We were unable to count the number of sperm stored in the paired spermathecae due to poor image quality; therefore, only counts from the seminal receptacle counts were used in our analysis as a proxy for sperm retained after ejection. In *D. melanogaster* females most sperm are stored in the seminal receptacle (Gilbert 1981; Schnakenberg et al. 2011; Zelinger et al. 2024), and raw data from previous studies (Sanghvi, Shandilya, et al. 2025) shows a strong correlation between sperm counts in the seminal receptacle and paired spermathecae ($R^{2}$ = 0.442). This likely minimised error in our study resulting from the omission of spermathecal sperm counts. All sperm counts were done using FIJI/ImageJ (Appendices 2 and 3).

### 2.3 Data Analysis

Overall sample sizes for each analysis ranged from 48 to 66 individuals (Table 1). All analyses were conducted using Rv4.3 (R core team 2020). We created four models to address our aims.

First, we used a Cox‐proportional hazards model in the *survival* package (Therneau 2001) to test how the probability of ejection within the observation window (1 or 0 as censoring status) and ejection latency (time‐to‐event, dependent variable) were impacted by the fixed effects of mating latency (Aim 1), male presence (Aim 2), and day. Next, for females that ejected the mating plug along with part of the ejaculate, we constructed two separate generalised linear models (GLM) with a negative binomial error distribution (to account for overdispersion in Poisson models), using the *glmmTMB* package (Brooks et al. 2017). We did not use Gaussian error distributions due to these being count data. Our GLMs tested whether: (i) the numbers of sperm ejected by females (*N*_*E*_) and (ii) the numbers of sperm retained by females in the seminal receptacle (*N*_*SR*_) were influenced by the fixed effects of mating latency, male presence, day and ejection latency. In these models, we included ejection latency to test whether females who ejected the mating plug earlier ejected more sperm or retained fewer sperm than females who ejected later (Aim 3).

Variation in the numbers of sperm ejected or retained by females could be a consequence of the number of total sperm transferred by the male, rather than merely represent female post‐mating ‘preference’ (Box 1). In our dataset, sperm numbers in the seminal receptacle were positively correlated with sperm numbers ejected (*R* ^2^ = 0.163, *β* = 0.07, *P*_lm_ = 0.003, Figure S1), indicating that females who were inseminated with more sperm, stored, as well as ejected more sperm. Due to this, we additionally tested how the *proportion* of sperm ejected (*P*_*E*_) was influenced by our variables of interest, to account for the differences in sperm number inseminated by males (Aim 3). For females that ejected part of the ejaculate, we constructed a GLM with a beta‐binomial error distribution to test how *P*_*E*_ was influenced by the fixed effects of mating latency, ejection latency, day and male presence. Data in this model were weighted by the total number of sperm counted (i.e., *N*_*E*_ + *N*_*SR*_). The proportion of sperm ejected was calculated as: $P_{E}=N_{E}/\left(N_{E}+N_{\mathrm{SR}}\right)$. As a sensitivity analysis, we tested whether including females that did not eject mating plugs altered the results. Findings from this model were consistent with our main analysis and are presented in Appendix 4.

We ensured model assumptions (overdispersion for GLM; constant hazard function for Cox) were not violated, using the *DHARMa* (Hartig and Hartig 2017), *performance* (Lüdecke et al. 2021) and *coxme* (Therneau 2001) packages.

## 3 Results

### 3.1 Mating Latency Versus Ejaculate Ejection

**FIGURE 2:**
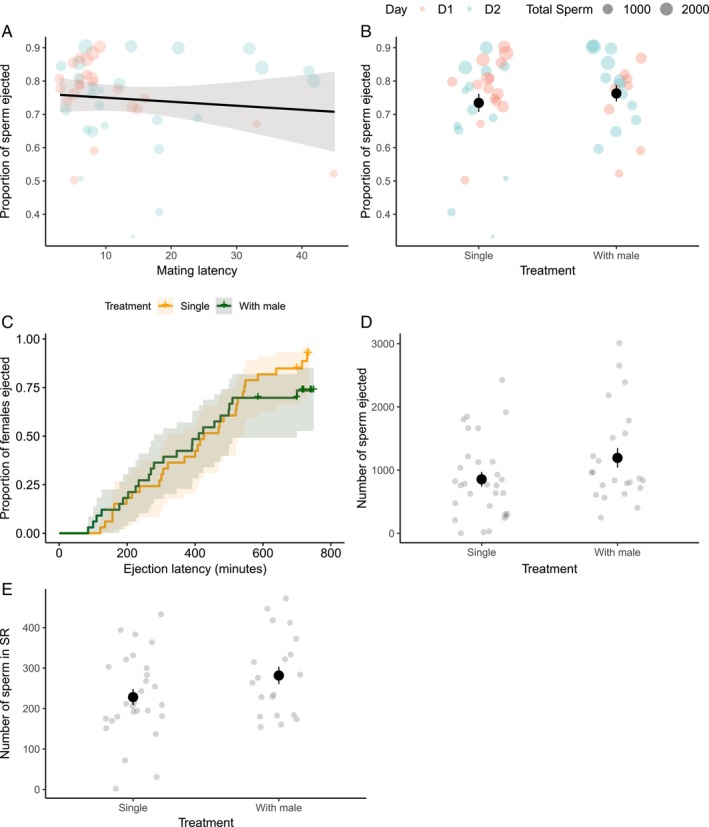
Influence of mating latency and male presence in ejection chamber, on different proxies for ejaculate ejection behaviour. (A) No correlation between mating latency and the proportion of sperm ejected by the female. No significant effect of male presence on (B) the proportion of sperm ejected; (C) ejaculate ejection latency; (D) the numbers of sperm ejected along with the mating plug; (E) the numbers of sperm stored in the female's seminal receptacle. Error bars show SEM, shaded areas show 95% CI. Red dots show data from Day 1, blue dots show data from Day 2. “+” in (C) represent 11 females who did not eject (right‐censored), and one female who died in the ejection chamber (left‐censored).

In Aim 1, we investigated whether pre‐ and post‐copulatory preference of females correlate with each other, to understand whether different episodes of sexual selection occur concurrently or disruptively. We found no significant correlation between mating latency and ejection latency (HR (Hazard ratio) = 1.003, *z* = 0.254, *p* = 0.800, Table S1), the numbers of sperm ejected ‘*N*_*E*_’ (*β*_standardised_ = −0.04, *z* = −0.330, *p* = 0.741, Table S2), the numbers of sperm retained ‘*N*_*SR*_’ (*β*_standardised_ = 0.06, *z* = 0.241, *p* = 0.809, Table S3), or the proportion of sperm ejected ‘*P*_*E*_’ (*β*_standardised_ = 0.04, *z* = −0.499, *p* = 0.618, Table S4, Figure 2A, Appendix 4).

### 3.2 Influence of Male Presence

In Aim 2, we tested whether the presence of a novel male in the female's post‐mating environment facilitated earlier ejaculate ejection or ejection of more sperm, given the benefits of polyandry to female fruit flies. Contrary to predictions, we found that the presence of a male in the female's post‐mating environment did not impact ejection latency (HR = 0.817, *z* = −0.736, *p* = 0.461), *N*_*E*_ (*β*_standardised_ = 0.24, *z* = 0.916, *p* = 0.359), *N*_*SR*_ (*β*_standardised_ = 0.46, *z* = 0.958, *p* = 0.338), or *P*_*E*_ (*β*_standardised_ = −0.10, *z* = 0.559, *p* = 0.576) (Figure 2B–E; Tables S1–S4, Appendix 4).

### 3.3 Proxies for Ejaculate Ejection

In Aim 3, we investigated whether the commonly used proxy of ejection latency correlated with other proxies of ejaculate ejection behaviour, to understand whether these are interchangeable and understand the biological meaning of earlier versus later ejection. Surprisingly, ejaculate ejection latency did not significantly correlate with other proxies of ejection behaviour, namely, *N*_*E*_ (*β*_standardised_ = −0.14, *z* = −1.214, *p* = 0.225), *N*_*SR*_ (*β*_standardised_ = 0.13, *z* = −1.584, *p* = 0.113), or *P*_*E*_ (*β*_standardised_ = −0.02, *z* = 0.269, *p* = 0.788).

## 4 Discussion

### 4.1 Mating Latency Versus Ejaculate Ejection

In Aim 1, we investigated the relationship between proxies for pre‐ and post‐mating sexual selection. We found no evidence for mating latency correlating with ejection latency, or with the number or proportion of sperm ejected or retained. Contrasting direction of pre‐ and post‐mating sexual selection is expected when underlying trade‐offs between investment in traits that improve mating success or expedite time to mating, and ejaculate traits that improve fertilisation success or sperm retention by females, exist (Arbuthnott 2018; reviewed in Evans and Garcia‐Gonzalez 2016; Sanghvi, Henshaw, et al. 2025). Such a negative relationship has been shown by Durrant et al. (2016) in hissing cockroaches, where males with larger weapons have smaller testes, and by Danielsson (2001) in water striders. Similarly, in fruit flies, males who are worse at acquiring mates also invest more in their ejaculates or gain higher paternity share (De Nardo et al. 2021; Filice and Dukas 2019; Von Hellfeld et al. 2025). On the other hand, pre‐ and post‐mating selection can occur in concert when females are consistent in their choice of partners at both stages, or when males who are in higher condition not only acquire mates easily but also produce larger or higher quality ejaculates.

The lack of association in our study suggests that sexual selection might be acting independently at pre‐ and post‐mating stages. This result also implies that proxies for pre‐mating sexual selection cannot be used to interpret the direction of post‐mating sexual selection. However, there are also methodological factors that may explain the lack of association. First, low sample sizes in our study could have increased the likelihood of type II errors. Second, keeping females as virgins for 7–11 days may have reduced their choosiness (Kohlmeier et al. 2021), thereby weakening any associations. Third, the absence of a competitive mating context may have reduced the biological relevance of mating and ejection latency as metrics of sexual selection (Lüpold et al. 2013). Doubovetzky et al. (2024) showed that, in fruit flies, female ejection latency depends on the interaction between mating latency and the male strain used. Thus, fourth, the specific fly strains used in our study, while appropriate for addressing our research aims, may have also contributed to the lack of association between mating and ejection latency. Fifth, in *D. melanogaster* , mated females accept copulations slower and show stronger mate choice than virgin females (Pavković‐Lučić and Kekić 2009). Thus, it is possible that the virgin females in our study mated indiscriminately with *dah* males, thereby weakening the relationship between pre‐ and post‐copulatory selection. Given that *D. melanogaster* females benefit from polyandry (Yan et al. 2025), future studies could compare the association between mating latency and ejaculate ejection behaviour for mated and virgin females. Sixth, the absence of food in the female's post‐mating environment might have created an ‘unnatural’ environment and possibly impacted her ejection behaviour.

The biological mechanisms influencing mating and ejection latency can be male‐ and/or female‐driven. In fruit flies, mating latency is frequently used as a proxy for female pre‐mating preference, in a competitive as well as non‐competitive context (e.g., Dewan et al. 2025; Dukas 2005; Hosken et al. 2008; Kohlmeier et al. 2021; Poissonnier et al. 2024; Robinson et al. 2012; Sanghvi, Shandilya, et al. 2025; Savic Veselinovic et al. 2017; Sharma et al. 2010; Vega‐Trejo et al. 2024). Similarly, ejaculate ejection latency is commonly used to quantify female post‐mating preference (e.g., Doubovetzky et al. 2024; Yun et al. 2024). However, these proxies can also be expressions of male‐driven traits rather than female preference (Pitnick and Brown 2000). For instance, males who are more coercive or better at harassing females are reported to have shorter mating latencies despite being less attractive (Løvlie and Pizzari 2007). Similarly, because sperm motility is essential for entry into female storage organs in flies (Holt and Fazeli 2016; Schnakenberg et al. 2012), males with less motile sperm may be more likely to have their sperm ejected, independent of any female‐mediated preference. These processes underscore the difficulty in disentangling female‐ and male‐driven mechanisms of pre‐ and post‐mating sexual selection (Firman et al. 2017; Lüpold et al. 2020; Pitnick and Brown 2000). We thus encourage researchers to make these assumptions clear when assigning agency to either sex when quantifying pre‐ and post‐mating sexual selection.

### 4.2 Influence of Male Presence

In Aim 2, we tested whether the presence of a male in the female's post‐mating environment influenced her ejaculate ejection behaviour. We predicted that female flies would eject ejaculates sooner in the presence of another male either to facilitate multiple mating and increase the genetic diversity of their offspring (Jennions and Petrie 2000), to gain additional seminal fluid (Sanghvi, Shandilya, et al. 2025; Sepil et al. 2020), or to increase their attractiveness to another male, which can be masked by pheromones in the mating plug (Laturney and Billeter 2016). However, we found no effect of male presence on ejaculate ejection behaviour, regardless of the proxy used to measure ejection.

There could be several explanations for this lack of effect and disagreement with results from recent studies. Doubovetzky et al. (2024) recorded ejaculate ejection latency in fruit flies in response to the presence of males from two different strains. They found that when males and females differed in strain, there was no difference in ejection latency between females kept in isolation and those kept with another male after mating. However, when the male present in the post‐mating environment was of the same strain, females ejected ejaculates sooner than when kept in isolation. In our experiment, the female and the competitor male were from different strains, which may explain the absence of an effect. Specifically, females may have perceived *sot* males as less attractive than *gfp* males, rendering their presence negligible. Conversely, *sot* males might have been worse at courtship compared to *gfp* males, therefore reducing the rate of ejaculate ejection. While previous studies have shown that *sot* males have similar mating latency and mating probability to *dah* and *gfp* males (Sepil et al. 2020; Sanghvi, Shandilya, et al. 2025), the differences in strains might have impacted our results in unexpected ways. Yun et al. (2024) also showed that a female fly will eject sperm sooner when she can smell male pheromones in her post‐mating environment. We manipulated male presence not through odour cues, but directly, thus we were unable to separate the perception of male presence from actual male presence. Therefore, in our study, factors such as harassment by the second males could have confounded the results. Discrepancy between our result and that of Yun et al. (2024) could also be explained if information from different modalities is conflicting, thus smell playing a greater role in mediating ejaculate ejection than modalities (Doubovetzky et al. 2024).

### 4.3 Proxies for Ejaculate Ejection

We explored how different proxies of female ejaculate ejection behaviour correlate, to understand whether they are interchangeable and capture the same biological meaning in the context of post‐mating sexual selection. Studies typically use ejection likelihood (Pizzari and Birkhead 2000), ejection latency (e.g., Lüpold et al. 2013; Manier et al. 2010; Yun et al. 2024), or absolute sperm numbers ejected or stored (e.g., Córdoba‐Aguilar 2006; Doubovetzky et al. 2024) as proxies for female post‐mating preference. However, few have tested whether these measures, thus their results, are comparable. These metrics can be confounded. For instance, if sperm from higher quality males reach storage faster, females may eject their sperm sooner simply because storage fills up earlier. Here, ejection latency would not indicate a preference for male quality, despite being interpreted as such. Similarly, if larger ejaculates result in more sperm being ejected (our results; also see Dean et al. 2011), then numbers of sperm ejected would reflect the overall quantity of sperm inseminated rather than female preference. We suggest that the proportion of sperm ejected or retained (*P*_*E*_ or 1−*P*_*E*_) be used in conjunction with other proxies such as ejection latency, ejection likelihood, numbers retained and numbers ejected. Proportion sperm ejected provides a biologically meaningful understanding of post‐mating sexual selection in contexts where other proxies might be confounded (Box 1). However, in species where sperm are degraded and absorbed by the female, some sperm inside female reproductive organs will avoid detection, and in such systems, *P*_*E*_ should be interpreted with caution as it is likely to be overestimated. To our knowledge, ours is the first study to empirically demonstrate the importance of using *P*_*E*_ to quantify post‐mating sexual selection.

In species like fruit flies with strong last male sperm precedence via sperm displacement, different proxies of ejaculate ejection might not necessarily correlate with male fitness (Laturney et al. 2018). Specifically, retaining greater numbers of sperm from a male might not directly translate to a greater paternity share of that male under sperm competition (Manier et al. 2010). Future research could investigate whether correlations between different proxies of ejaculate ejection behaviour are modulated by sperm competition by remating females. Specifically, studies could test whether later ejection, greater sperm retention and lower sperm ejection of the first male translate to a greater paternity share under sperm competition.

## 5 Conclusions

The relationship between pre‐ and post‐mating sexual selection is complex. We used various proxies of ejaculate ejection behaviour to quantify post‐mating sexual selection and found no association between pre‐ and post‐mating sexual selection, irrespective of which proxy was used. Such decoupling of selection can potentially explain the maintenance of variation in sexually selected traits. In contrast to some studies, we found no effect of male presence in the female's post‐mating environment on ejaculate ejection behaviour. This discrepancy could be due to methodological differences between our work and previous studies. Importantly, we provide biological reasons for using multiple proxies of female ejaculate ejection behaviour rather than relying on a single metric and propose that ‘proportion of sperm ejected’ should be included as a complementary measure. Our results have broad implications for understanding and measuring sexual conflict, sexual selection and reproductive strategies in animals.

## Author Contributions

**Krish Sanghvi:** conceptualization (equal), data curation (equal), formal analysis (equal), investigation (equal), methodology (equal), software (equal), validation (equal), visualization (equal), writing – original draft (equal). **Biliana Todorova:** validation (equal), writing – review and editing (equal). **Irem Sepil:** investigation (supporting), supervision (lead), writing – review and editing (equal).

## Conflicts of Interest

The authors declare no conflicts of interest.

## Supporting information


**Figure S1:** ece372281‐sup‐0001‐Supinfo.docx.

## Data Availability

All data from our experiment and the R code used for analysis can be found at OSF under an anonymous link: https://osf.io/gdhyq/files/osfstorage?view_only=689704dac2b54656bd66f9c5be293f74. Images of dissected females' SR and their ejected ejaculates can be found on Figshare: https://doi.org/10.6084/m9.figshare.28164290.v1; https://doi.org/10.6084/m9.figshare.28164263.v1.

## Appendix 1 STRANGE Framework

### Social Background

Parents of experimental individuals had been kept in mixed‐sex, freely mating populations cages in a temperature and humidity‐controlled lab. Experimental males were kept as virgins in single‐sex vials of 10 individuals and did not interact with individuals of the opposite sex until the mating assay.

### Trappability and Self‐Selection

Experimental flies were reared using a random subset of eggs acquired from the population cages, laid by stock flies in those cages. Post‐eclosion, a random subset of flies was collected as virgins for our experiment.

### Rearing History

Experimental males only interacted with other virgin experimental males in groups of 10 until the mating assay. They were kept in transparent plastic vials with food and without any other objects. Experimental females were kept singly.

### Acclimation

Flies were kept in vials for 7–11 days before being used for assays.

### Natural Changes in Responsiveness

The lab was 24/7 temperature controlled at 25°C and on a 12:12 h light–dark cycle.

### Genetic Makeup

Experimental males of *gfp* background had transgenes that expressed green fluorescent protein in sperm heads and had been reared in our lab for at least the past 8 years. These flies had been previously backcrossed in *dah* background. For most of this time, *gfp* flies were reared at 20°C; however, for ~4 generations prior to the experiment, they were maintained at 25°C. Females were from outbred *dah* strain and were derived from populations of wild‐type flies collected in Benin (Africa) and maintained in our lab since the 1970s at 25°C. *sot* males were obtained by crossing two parental lines (Tudor and *dah*) following Sanghvi, Henshaw, et al. (2025), Sanghvi, Shandilya, et al. (2025), Sanghvi, Gascoigne, and Sepil (2025) and only persist for a single generation due to being infertile.

### Experience

Experimental flies had not participated in any previous assays prior to this study.

## Appendix 2 Sperm Counts in Ejected Ejaculate

We pipetted 4 μL of PBS on the ejected ejaculate in the ejection chamber. The mating plug along with the ejected sperm was collected using minutien 0.1 mm micropins while being observed under 20× magnification via a dissecting microscope. It was then transferred immediately onto a dry slide previously coated with a solution of gelatine (10 g/1 L water) and chromium potassium sulphate (2 g/1 L water). A coverslip was placed on the slide thereafter. The sperm present in the ejected ejaculate was carefully spread out using microneedles to improve visualisation. The sides of the coverslip were glued using rubber cement to prevent desiccation. On the following day, these sperm were imaged using a confocal Leica stellaris microscope (20× objective, 10× eyepiece magnification) through a GFP channel (excitation wavelength: 480–520 nm, gain: 230–260 units, pixel size: 1024 × 1024, scan speed: 200, pinhole: 1 AU), as well as with a T‐PMT channel (white light). The number of fluorescent sperm heads was later estimated automatically using the find maxima plugin on FIJI/ImageJ version win32 (Schindelin et al. 2012). The prominence on the find maxima plugin was set at 65 because this is where the power function relationship between prominence values and estimates of sperm number became linear and flat (following Sanghvi, Henshaw, et al. 2025; Sanghvi, Shandilya, et al. 2025; Sanghvi, Gascoigne, and Sepil 2025). Sperm counts done manually on images of ejected sperm were highly repeatable with sperm counts estimated automatically using the find maxima plugin (*R* ^2^ = 0.984 based on 16 images), therefore ensuring accuracy of automated sperm counts. Additionally, sperm counts done manually on 16 images of ejected sperm were highly repeatable between two analysts (*R* ^2^ = 0.99).

## Appendix 3 Sperm Counts in Seminal Receptacle

We dissected females using Inox Biology forceps, by pulling apart their last segment from the rest of the body to separate their reproductive tracts. Female reproductive tracts were then placed on a slide coated with gelatine and chromium potassium sulphate. The seminal receptacle and spermathecae were spread to allow clear imaging. Then, a coverslip was placed on the sample, and the sides of the coverslip were glued using rubber cement to prevent desiccation. The seminal receptacle, bursa and spermathecae on these slides were imaged on the same day of dissections, using a Nikon Eclipse50i fluorescence microscope (magnification = 10× objective, 10× eyepiece, wavelength = 480 nm) with a chromix HD camera, under UV light from a CoolLED pe300 light source. The number of sperm heads (which appeared as fluorescent green under UV light) was later counted manually from images, using the cell counter plugin on FIJI/ImageJ version win32 (Schindelin et al. 2012). Sperm counts were highly repeatable between two different analysts (*R* ^2^ = 0.98 across 11 images). Sperm images of the spermathecae were unclear, leading to inaccurate counts; therefore, only data on sperm stored in seminal receptacles were used.

We additionally dissected all females that had not ejected the ejaculate at the end of the ejection assay to check if these females had their mating plugs intact. This was done to ensure that their lack of ejection was not merely a detection error. All females that were recorded as not having ejected ejaculates indeed had their mating plugs intact (Figure 1E,F).

## Appendix 4 Sensitivity Analysis

We ran a model to understand how the proportion of sperm ejected was influenced by different variables, but here included not only females that ejected ejaculates, but also those that did not eject the ejaculate (*N*_total_ = 60). This was done to test whether our main model was sensitive to this inclusion criteria. For females that did not eject, we coded the proportion of sperm ejected (*P*_*E*_) as 0. We created a quasi‐binomial GLM with *P*_*E*_ as the dependent variable, and day, male presence, and mating latency as fixed effects. We did not include ejection latency in this model because females that did not eject were right‐censored for this variable. Like our main model, this analysis showed that the proportion of sperm ejected by females did not correlate with mating latency (*z* = 0.353, *p* = 0.725) and was not influenced by the presence of a male in her post‐mating environment (*z* = −1.653, *p* = 0.104).
