# Supplementary material for: Ejaculate Ejection by Female Fruit Flies Does Not Correlate With Mating Latency or Male Presence, Irrespective of How Ejection Is Measured
Source: Ecol Evol. 2025 Oct 7;15(10):e72281. doi: 10.1002/ece3.72281 (PMC12502626; doi:10.1002/ece3.72281)
Supplement: Supplementary file 1 — Figure S1: ece372281‐sup‐0001‐Supinfo.docx. [file ECE3-15-e72281-s001.docx]

Supporting information

**Figures**


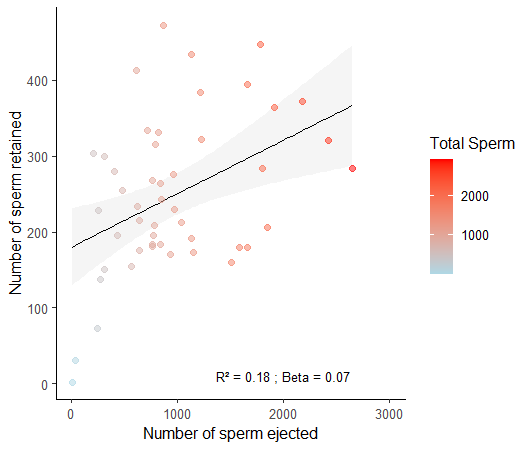


Figure S1: Numbers of sperm ejected by females correlates positively with the number of sperm retained by females, likely due to substantial variation in total sperm numbers transferred by males.

**Tables**

Table S1: Influence of mating latency (used as a proxy for pre-mating sexual selection), presence of male, and day, on ejection latency. Cox proportional hazards model where time-to-event represent ejection latency, censoring represents ejection probability (N = 66). Model $R^{2}$= 1.310%.

| Fixed effects | coef | exp(coef) | se(coef) | z | P |
| --- | --- | --- | --- | --- | --- |
| Day2 | 0.077 | 1.080 | 0.275 | 0.280 | 0.779 |
| Treatment (With male) | -0.202 | 0.817 | 0.275 | -0.736 | 0.461 |
| Mating latency | 0.003 | 1.003 | 0.014 | 0.254 | 0.800 |

Table S2: Influence of mating latency, presence of male, day, and ejection latency, on the numbers of sperm ejected (*N_E_*), in our ejaculate ejection assay. Model constructed as GLM with negative binomial error distribution (N = 54). Model $R^{2}$= 6.481%.

| Fixed effects | Estimate | SE | z | P |
| --- | --- | --- | --- | --- |
| (Intercept) | 7.118 | 0.350 | 20.349 | <0.001 |
| Mating latency | -0.004 | 0.011 | -0.330 | 0.741 |
| Ejection latency | -0.001 | 0.001 | -1.214 | 0.225 |
| Day2 | 0.021 | 0.260 | 0.081 | 0.936 |
| Treatment (With male) | 0.237 | 0.259 | 0.916 | 0.359 |

Table S3: Influence of mating latency, presence of male, day, and ejection latency, on the numbers of sperm stored in the female’s seminal receptacle (*N_SR_*) after her ejecting the ejaculate. Model constructed as GLM with negative binomial error distribution (N = 53). Model $R^{2}$= 8.689%.

| Fixed effects | Estimate | SE | z | P |
| --- | --- | --- | --- | --- |
| (Intercept) | 5.712 | 0.228 | 25.034 | <0.001 |
| Mating latency | 0.002 | 0.007 | 0.241 | 0.809 |
| Ejection latency | -0.001 | 0.000 | -1.584 | 0.113 |
| Day2 | -0.052 | 0.158 | -0.328 | 0.743 |
| Treatment (With male) | 0.155 | 0.162 | 0.958 | 0.338 |

Table S4: Influence of mating latency, presence of male, day, and ejection latency, on the proportion of inseminated sperm ejected by the female (*P_E_*) along with the mating plug, for females that ejected the ejaculate. Proportion of sperm ejected calculated as numbers retained in seminal receptacle, divided by the sum of numbers retained in seminal receptacle plus numbers ejected: *P_E_* = *N_SR_*/*(N_SR_* + *N_E_*). Model constructed as GLM with beta-binomial error distribution with *N_SR_* + *N_E_* included as weights (N = 48). Model $R^{2}$= 1%.

| Fixed effects | Estimate | SE | z | P |
| --- | --- | --- | --- | --- |
| (Intercept) | 1.093 | 0.261 | 4.180 | <0.001 |
| Mating latency | -0.004 | 0.008 | -0.499 | 0.618 |
| Ejection latency | -0.000 | 0.001 | 0.269 | 0.788 |
| Day2 | -0.032 | 0.177 | -0.178 | 0.859 |
| Treatment (With male) | 0.098 | 0.175 | 0.559 | 0.576 |
